# Enteric Methane Emission from Cattle Grazing Systems with Cover Crops and Legume–Grass Pasture

**DOI:** 10.3390/ani14233535

**Published:** 2024-12-07

**Authors:** José Ignacio Gere, Silvina Beatriz Restovich, Juan Mattera, María Isabel Cattoni, Abimael Ortiz-Chura, Gabriela Posse, María Esperanza Cerón-Cucchi

**Affiliations:** 1 Unidad de Investigación y Desarrollo de las Ingenierías, Facultad Regional Buenos Aires, Universidad Tecnológica Nacional, Ciudad Autónoma Buenos Aires C1179AAQ, Argentina; 2 Consejo Nacional de Investigaciones Científicas y Técnicas (CONICET), Ciudad Autónoma Buenos Aires C1033AAJ, Argentina; 3 Estación Experimental Agropecuaria Pergamino, Instituto Nacional de Tecnología Agropecuaria (INTA), Ruta 32 km 4.5, Pergamino B2700XAC, Argentina; restovich.silvina@inta.gob.ar (S.B.R.); mattera.juan@inta.gob.ar (J.M.); cattoni.maria@inta.gob.ar (M.I.C.); 4 UMR 1213 Herbivores Unit, Université Clermont Auvergne, INRAE, VetAgro Sup, F-63122 Saint-Genès-Champanelle, France; abimael.mpa.uba@gmail.com; 5 Instituto de Patobiología Veterinaria (IPVet), Instituto Nacional de Tecnología Agropecuaria (INTA-CONICET), Hurlingham C1417AZE, Argentina; 6 Instituto de Clima y Agua, Instituto Nacional de Tecnología Agropecuaria (INTA), Hurlingham C1417AZE, Argentina; posse.gabriela@inta.gob.ar

**Keywords:** beef cattle, grazing systems, agriculture, livestock production, sustainability, SF_6_ tracer technique

## Abstract

This study aims to quantify enteric methane (CH_4_) emission and dry matter intake (DMI) in beef steers under two rotational grazing systems: (i) a mixture of cover crops (vetch + ryegrass + forage radish) (CC) and (ii) alfalfa and fescue pasture (AFP). Eighteen Hereford steers were divided into two groups (nine steers per group), assigned to either the CC or AFP. Methane emissions were measured using the SF_6_ tracer technique. The results showed that steers grazing CC produced 29% less CH_4_ in g/d compared to those on the AFP (119.1 vs. 167.1 g/d for CC and AFP, *p* < 0.05) and 36% less CH_4_ yield (4.3 vs. 6.7% of gross energy intake). However, average daily gain (ADG), DMI, and CH_4_ intensity (gCH_4_/kg ADG) did not significantly differ between treatments. The integration of CC in a cattle grazing system has the potential to reduce CH_4_ emissions by improving forage quality.

## Simple Summary

This study examines the impact of different grazing systems on methane (CH_4_) emission and dry matter intake (DMI) in beef steers. It compares two systems: (i) a cover crop mixture (CC) and (ii) alfalfa and fescue pasture (AFP). The results showed that steers on CC produced 29% less methane (expressed in g/d) and 36% less CH_4_ yield (expressed as % of gross energy intake) than those on the AFP. However, the DMI, average daily gain, and CH_4_ intensity were similar between the two systems.

## 1. Introduction

Methane (CH_4_) is a potent greenhouse gas (GHG) with a global warming potential approximately 28 times greater than that of carbon dioxide (CO_2_) [1]. In livestock production, CH_4_ emissions account for about one-third of all anthropogenic CH_4_ emissions globally, primarily originating from ruminants, especially cattle, given their population size and substantial body mass [2]. Enteric CH_4_ is produced under anaerobic conditions in the rumen by methanogenic archaea, which convert CO_2_ and hydrogen into CH_4_, subsequently releasing it into the atmosphere [3]. Importantly, enteric CH_4_ emissions represent an energy loss ranging from 2 to 12% of the gross energy intake of ruminants [4]. Therefore, reducing enteric CH_4_ emissions has dual benefits: decreasing GHG emissions while improving production efficiency. Identifying effective CH_4_ mitigation strategies that do not compromise animal performance is particularly urgent in Latin American countries where livestock plays a critical role in supporting rural livelihoods.

In Argentina, pastures constitute a vital forage resource within livestock production systems, ranking as the third most cultivated resource after oilseeds and cereals, covering approximately 8 million hectares nationwide [5]. Pastures offer multiple benefits, including consistent feed quantity and quality at a lower cost than alternatives like forage reserves and supplements. Mixed grass–legume pastures are among the primary forage resource, representing 30% of the perennial pastures planted in the country [5]. Beyond forage production, effective pasture management also serves as a strategy for soil conservation, with improvements in soil organic carbon and nitrogen stocks and physical properties observed following the establishment of perennial pastures [6,7].

Integrating crop–livestock systems through effective management practices offers a promising approach to mitigating GHG emissions while enhancing sustainability. These integrated systems create diversified agroecosystems that contribute to ecological intensification by increasing food production, maintaining or improving environmental quality, and conserving natural biodiversity [8]. Growing concerns over the environmental impacts of conventional agroecosystems have led to increased interest in alternative cropping systems that enhance ecosystem multifunctionality [9]. In this context, increasing cropping intensity using cover crops or perennial pastures has been recommended as a rapid climate mitigation strategy for carbon sequestration [10,11], as well as for nitrogen conservation and recycling within the soil–plant system [12,13]. Cover crops and pastures also improve soil structure, reduce erosion, and enhance overall system sustainability while yielding crop production outcomes comparable to single-crop systems [14,15,16]. Additionally, grazing cover crops can provide further nutrient cycling and economic benefits to producers through increased livestock weight gain [17].

The nutritive value of forage varies significantly across forage types, including annual and perennial grasses, legumes, and tropical and temperate species [18]. Differences also exist between species and cultivars within species. Optimizing cattle nutrition involves selecting high-quality forage species and cultivars adapted to specific farm environments, balancing forage quantity and quality. Including legumes in forage mixtures has been shown to reduce CH_4_ emissions due to their lower fiber content, increased dry matter intake (DMI), and faster rumen passage rate [19]. Additionally, the high protein content and dry matter digestibility (DMD) of legumes make them a preferred choice in grazing systems [20]. Despite these developments, knowledge gaps remain, particularly regarding CH_4_ emission quantification from diverse forage types, including cover crops, under real-world grazing conditions. While cover crops are recognized for their role in soil conservation and carbon sequestration, limited data exist on their specific effects on enteric CH_4_ emissions by grazing cattle. Addressing these gaps is essential for developing targeted mitigation strategies and enhancing the environmental sustainability of livestock production.

Therefore, this study aims to assess enteric CH_4_ emissions, DMI, and average daily gain (ADG) in beef steers under two grazing systems: a cover crop mixture and a legume–grass pasture.

## 2. Materials and Methods

### 2.1. Study Site

A field experiment was set up in July–August 2021 at the Pergamino Experimental Station of the Instituto Nacional de Tecnología Agropecuaria (INTA, Argentina) (33°51′ S, 60°40′ W). Soil type is a Typic Argiudoll (USDA Soil Taxonomy) of the Pergamino series with a silt loam A horizon without eroded phase (<0.3% slope) and a strong argillic B horizon. The climate in the study area is temperate humid, without a dry season, with a mean annual temperature of 16.5 °C and mean annual rainfall of 984 mm for the 1910–2024 period (Agroclimatological network database, INTA). Rainfall and average temperature during the month (August) in which this study was carried out was 26.4 mm and 12.1 °C, respectively.

### 2.2. Animals and Experimental Design

Eighteen Hereford steers aged 12 to 15 months and weighing 244 ± 18 kg (average weight ± standard deviation) were randomly divided into two groups based on initial body weight:

(i) A mixture of cover crops was integrated into a soybean–maize sequence (CC). The CC was shown non-till on 23 April 2021, and the species used as cover crops were annual ryegrass (*Lolium multiflorum* Lam.), hairy vetch (*Vicia villosa* L.), and a forage radish (*Raphanus sativus* L.), with densities of 13, 30, and 2 kg of seed/ha, respectively;
(ii) Alfalfa (*Medicago sativa* L.) and fescue (*Lolium arundinaceum*) pasture (AFP), with densities of 12 and 8 kg of seed/ha, respectively.

**Figure 1:**
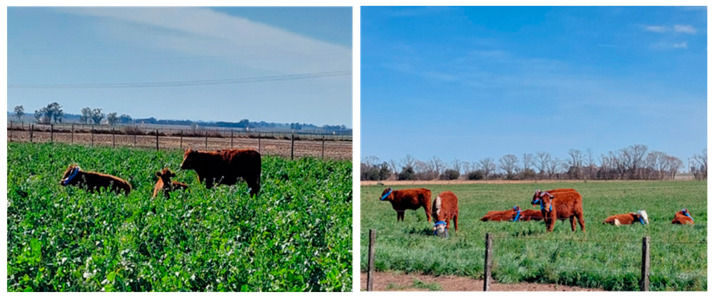
Images of the two systems evaluated: a cover crop mixture with annual ryegrass, hairy vetch, and a forage radish (CC) (**left**); and an alfalfa-fescue pasture (AFP) (**right**).

Both CC and AFP were sown as a mixture of the species involved in the same area (Figure 1). Before the trial, the animals underwent a 15 d acclimatization period to adjust to the established diet. The steers were allowed to freely graze on any of the species offered in paddocks of 0.5 ha, divided using electric fences (two paddocks of CC and one paddock of AFP). Drinking water was readily available. Both cover crops and pasture were grazed in a rotational system, with a grazing period of 10 d followed by a resting period of 20 d. Forage allocation was set at 3% of the average body weight to adjust the stocking rate.

The animals were weighed on three opportunities (0, 15, and 43 d), with a 16 h fasting period prior to each weighing. ADG during the experiment was calculated by dividing the weight difference by the 28-d interval between them.

The protocols, procedures, and animal care were approved by the Institutional Committee for the Care and Use of Animals (CICUAE File No. 34/21, approval date 15 September 2021, INTA).

### 2.3. Herbage Measurement

Forage mass measurements were taken before and after grazing in each paddock. Three randomly allocated quadrats of 100 cm × 100 cm were used to collect forage samples, by cutting at a height of 5 cm, which were pooled from each paddock of each treatment. Additionally, another sample from each paddock was separated into hairy vetch, annual ryegrass, forage radish, alfalfa, tall fescue, weeds, and dead material, according to the treatment. Both herbage mass and pasture botanical composition were dried in a forced air oven at 60 °C for 48 h and expressed on a dry weight basis.

All of the dried samples, cover crops, and AFP were ground using a Wiley Mill to pass through a 1 mm mesh and stored until further chemical composition analysis. Dry matter (DM) content was determined by drying samples at 105 °C for 24 h, while ash content was assessed by incinerating samples in a muffle furnace at 550 °C for 4 h, in accordance with method 942.05 [21]. Total nitrogen (TN) was measured using the Kjeldahl method as outlined by method 46–129 [22], with crude protein (CP) calculated as TN × 6.25. Neutral detergent fiber (NDF) and acid detergent fiber (ADF) contents were sequentially analyzed using an Ankom 220 fiber analyzer (ANKOM Technology Corporation, Fairport, NY, USA), following the procedures described by Van Soest et al. [23]. Gross energy (GE) was measured using a bomb calorimeter (PARR 1261, Parr Instrument Company, Moline, IL, USA). Dry matter digestibility was estimated using the equation derived from the FDA percentage [24].

### 2.4. Enteric Methane Measurement

**Figure 2:**
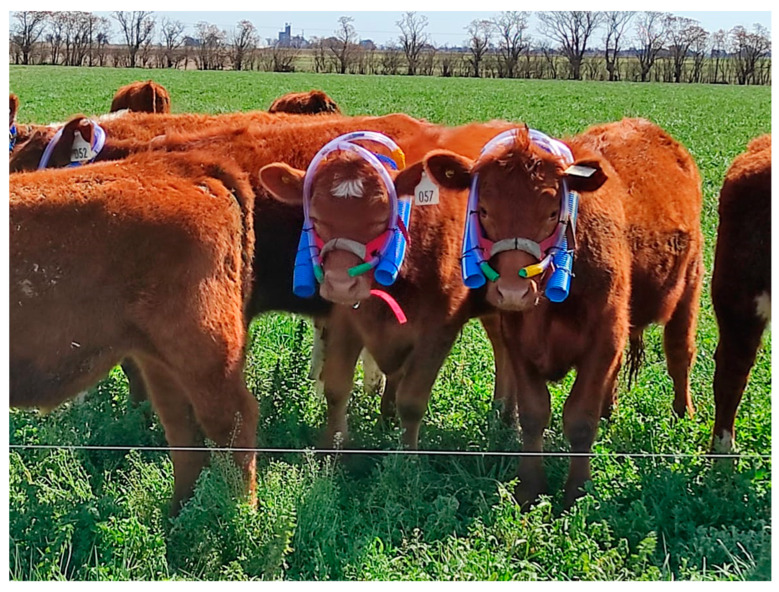
Animals in the experiment with equipment set up for monitoring enteric methane emissions using the SF_6_ tracer technique. Two sample collection systems are placed for each animal inside the blue corrugated tube, which serves to contain the equipment, and the tube is secured to the muzzle.

The enteric CH_4_ emissions were measured using the sulfur hexafluoride (SF_6_) tracer gas technique [25]. A brass permeation tube, with a known permeation rate of SF_6_ (average 8.87 ± 1.88 mg/d), was orally administered to steers 20 d before the beginning of the collection period. The sample collection system comprised two steel vessels (0.5 L volume), and the sample flow regulator consisted of a metal capillary (10 cm length), with a small section (5 mm) pressed until the desired flow rate of 0.05 mL/min was achieved. A target internal pressure of approximately 500 mbar (±100 mb) was maintained in the collection device at the end of the sample collection period. The sampling duration lasted for 5 consecutive d, following the recommendation by Gere and Gratton [26] and Pinares-Patiño et al. [27] (Figure 2). Background air samples were also collected using the same sample collection systems positioned at grazing height at a distant site, away from the animal location, to establish the baseline atmospheric concentrations of CH_4_ and SF_6_. These background samples were collected in duplicate, oriented in the direction of the prevailing wind. The concentrations of CH_4_ and SF_6_ were analyzed at the Pathobiology Veterinary Institute (CICVyA, INTA) using a gas chromatograph (Perkin Elmer 600, Kansas City, MO, USA), following the methodology described by Gere et al. [28].

### 2.5. Dry Matter Intake

The method involved indirectly measuring the dry matter intake (DMI) of animals by using titanium dioxide (TiO_2_) as an external marker, a technique outlined by Short et al. [29]. Animals were administered gelatin capsules containing 10 g of TiO_2_ (99% purity) each morning for 10 d. This TiO_2_ regimen was initiated 5 d before the sampling period to establish ruminal equilibrium. Fecal samples were collected over the next 5 d via rectum post-TiO_2_ administration within a 30 min timeframe. These samples underwent a series of processing steps, including drying at 60 °C for 96 h and grinding. Composite fecal samples were created from 2 g portions collected over 5 d for each animal and were subsequently analyzed for various components such as NDF, ADF, and TiO_2_ concentration [30]. The mean daily DMI per animal was then calculated based on the fecal dry matter output (DMf) and the dry matter digestibility (DMD %) of consumed herbage, following the approach detailed by Corbett and Freer [31] (Equation (1)).

(1) $$DMf\;(kg/d)=TiO_{2}\;dose\;(mg/d))/(TiO_{2}\;in\;faeces-TiO_{2}\;in\;feed\;(mg/kg\;DM))$$

The daily DMI per animal was calculated using the DMf and the DMD, as shown in Equation (2):

(2) $$DMI\;(kg/d)=\frac{DMf\left(kg/d\right)\times 100}{100-DMD\times 100}$$

where the DMD was estimated using the indigestible NDF content as the internal marker for both the diets and the feces, as described by Schalla et al. [32].

### 2.6. Data Analyses

The Infostat Statistical Software (Infostat 2020) [33] was utilized to analyze differences in the mean values of DMI, ADG, CH_4_ emissions, emission intensity (CH_4_/kg ADG), and CH_4_ yield expressed per kg DMI, or as a percentage of the ingested GE (Ym) using ANOVA and Fisher’s LSD Test according to a linear mixed model represented by Equation (3):

(3) $$Y_{ij}=µ+T_{i}+E_{ij}$$

where Y_ij_ is the dependent variable, µ is the general mean, Ti is the fixed effect of the treatment (i = CC and AFP), and E_ij_ is the residual error. Origin Lab 6.0 software (OriginLab Corporation 2016) was used to calculate the slopes of the linear regressions for CH_4_/kg ADG versus ADG.

## 3. Results and Discussion

### 3.1. Forage Mass Production of Cover Crops and Legume–Grass Pasture

The results of forage mass production, based on dry weight, are presented in Table 1. The production of offered forage was comparable between CC and AFP (*p* = 0.298). The dry matter content of forage was higher in AFP than CC, both in pre-grazing offered forage (*p* = 0.006) and in residual post-grazing forage (*p* = 0.004). The differences can be attributed to the botanical composition; in the AFP treatment, a substantial portion of dead material was present in the offered herbage mass, while in the CC treatment, dead material was negligible. Dead material contributes to an increase in the dry matter content of forage.

### 3.2. Chemical Composition of Cover Crops and Legume–Grass Pasture

Both treatments showed favorable values in chemical composition variables (Table 2); however, the nutritive value of the forage was significantly higher (*p* ≤ 0.05) in the CC treatment compared to AFP, as evidenced by a higher CP content, greater dry matter digestibility (DMD), and lower fiber content. These differences in chemical composition can be attributed to the higher proportion of legumes in CC relative to AFP (28.5% vs. 22.9%), as legumes generally contain higher CP levels. The results align with the botanical composition of each treatment: CC contained a larger proportion of green live material, whereas AFP had a significant amount of dead material (Table 1), which likely reduced the nutritive value of its forage mass. These differences between the two resources, with CC being annual and AFP perennial, are particularly evident during the winter season. The CC exhibited more active growth, accumulating green biomass with minimal dead material, while AFP showed slower growth, leading to a higher accumulation of dead material, particularly in alfalfa, which is more active in spring and summer but susceptible to frost. Six instances of agronomic frost occurred in the two weeks preceding grazing, which further contributed to decline alfalfa in AFP.

Despite the rotational grazing system employed for AFP, it was insufficient to prevent the build-up of senesced material. Additionally, forage maturity directly impacts nutritive value due to physiological and phenological changes within the plant. As plants mature, their dry matter production generally increases; however, this growth is often accompanied by a decline in digestibility and CP content [34]. While increased maturity typically reduces forage quality, several environmental and agronomic management factors can modify the relationship between plant maturity and forage quality [35].

### 3.3. Animal Performance: Averaged Daily Gain and Dry Matter Intake

The average ADG and DMI did not differ significantly between treatments (*p* > 0.05) (Table 3). Overall, the animals demonstrated good performance in terms of ADG during the study. While the 28-d study period provided a useful indicator of weight gain, this timeframe may have been too short to capture any significant differences in weight gain between treatments. The ADG values observed in our study were slightly higher than those reported in previous research. Marín et al. [36] reported average daily gains of 0.74 kg/d for low residual feed intake (RFI) and 0.67 kg/d for high-RFI Hereford heifers, with an average body weight of 270 kg, grazing on natural pastures. The lower gains in their study were likely due to the forage’s lower quality, which had a CP content of 8% and higher fiber levels (NDF: 71%, ADF: 44%).

Although higher-quality feed is generally associated with increased weight gain at comparable DMI levels, other factors—such as phenotypic variation, digestion and rumen fermentation efficiency, and nutrient partitioning—may limit this effect [37]. Consequently, improvements in diet quality do not always translate to increased weight gain. The similar ADGs observed between steers grazing on AFP and CC, despite differences in DMD, may be attributed to selective grazing behavior in AFP. This behavior likely allowed animals to avoid senesced material, effectively increasing the DMD of the forage they consumed.

The high performance observed in the animals in our study may be explained by the well-documented potential of legume pastures to enhance beef cattle production in both tropical and temperate regions [38,39,40]. Due to their high protein content resulting from distinct photosynthetic pathways, legumes provide a sustainable and cost-effective protein source for cattle grazing on low-quality forage. Regarding DMI, our results are in agreement with those of Marín et al. [36], who reported intakes of 7.16 kg/d and 6.78 kg/d for low- and high-RFI heifers, respectively, in grazing conditions without supplementation (8% CP, 71% NDF, 44% ADF). Similarly, Dini et al. [41] observed DMI values of 9.33 kg/d and 10.6 kg/d for low- and high-RFI steers, respectively, with animals feeding twice a day with a fully mixed ration (13% CP, 48% NDF, and 31% ADF).

In terms of the productive efficiency of cover crops, research conducted at Auburn University found that, under a put-and-take grazing system, yearling steers (266 kg BW) grazing on a mixture of cereal rye, oat, crimson clover, and a turnip × rapeseed hybrid achieved an ADG of 1.1 to 1.3 kg/d [42], results that are comparable to those reported in this study. Additionally, similar findings (1.1 kg/d) were reported by Planisch et al. [43] for beef steers grazing on annual ryegrass in an integrated ryegrass–soybean rotation system.

### 3.4. Methane Emissions

Significant differences in daily CH_4_ emissions were observed, with CC showing lower emissions compared to AFP (119.1 vs. 167.1 g/d; *p* = 0.0018), representing a 29% reduction for CC. Since no differences were found in DMI, this reduction persisted when calculating CH_4_ yield, whether expressed per kg of DMI or as a percentage of ingested GE (Ym). However, no differences were noted in CH_4_ intensity expressed in g CH_4_/kg ADG (Table 3).

The CH_4_ emission values observed in this study are consistent with previous research in extensive beef cattle systems, particularly with animals of similar characteristics in grazing conditions. In the Pampas region of Argentina, Bárbaro et al. [44] reported CH_4_ emissions of 162–167 g/d in Aberdeen Angus steers with an average body weight of 265 kg, while Gere et al. (2019) [28] found emissions ranging from 157 to 203 g/d in Aberdeen Angus × Hereford cows weighing around 380 kg. Gonzalez et al. [45] also recorded emissions between 131 and 251 g/d in Aberdeen Angus heifers with body weights ranging from 360 to 450 kg.

Although CH_4_ emissions (g/d) were lower in CC than AFP, DMI did not differ between treatments. Several studies suggest that the level DMI is a more significant factor than pasture quality in determining absolute CH_4_ emissions [46,47]. However, the difference in absolute CH_4_ emissions in the current work cannot be explained only by differences in DMI. Improving forage quality has been suggested as a potential method to mitigate CH_4_ emissions from ruminants [48,49,50]. For this reason, differences in CH_4_ emissions were explained by changes in nutritive value of the pastures. For instance, Gaviria-Uribe et al. [51] reported that DMI and diet composition have a significant impact on enteric CH_4_ production. This experiment examined different levels of intensification in cattle production systems, including naturalized pastures, improved pastures, and silvopastoral systems. Diets incorporating *Leucaena* forage legumes generally provided higher CP levels and increased DMI. The inclusion of *Leucaena* improved nutrient intake, resulting in greater ADGs in cattle. Consequently, the emission intensity from legume-based systems was lower, indicating that these systems could be an effective option for meeting emission reduction targets in sustainable tropical cattle production. Many CH_4_ emission reduction strategies focus on managing these two components, which align with our own observations. Diets with higher nutritional quality—characterized by increased digestibility, higher CP content, and lower NDF and ADF levels—resulted in higher DMI and reduced CH_4_ emissions, thereby minimizing energy loss in the form of CH_4_ (Ym) [51].

The emission reductions observed in this study (approximately 30% in absolute values for g CH_4_/d and Ym in %) are too substantial to be explained solely by the improved forage quality found in cover crops. It has been demonstrated that incorporating specific forage species can reduce enteric CH_4_ emissions. Dillard et al. [52] found that cattle diets containing *Brassica* spp. led to lower CH_4_ production per day, per gram of digestible organic matter, and per gram of DNF when evaluated in a continuous culture fermenter system. This suggests the presence of an additional mitigating factor, potentially related to secondary metabolites. Plants produce secondary metabolites, such as tannins, saponins, and essential oils, which can have toxic effects on bacteria, protozoa, and methanogenic archaea. These effects alter bacterial and protozoan populations, which, due to their commensal relationships, indirectly reduce methanogenic archaea populations, ultimately leading to lower CH_4_ production [53]. However, it is also known that alfalfa-based pastures contain secondary metabolites that can contribute to the mitigation of enteric CH_4_ emissions [54].

**Figure 3:**
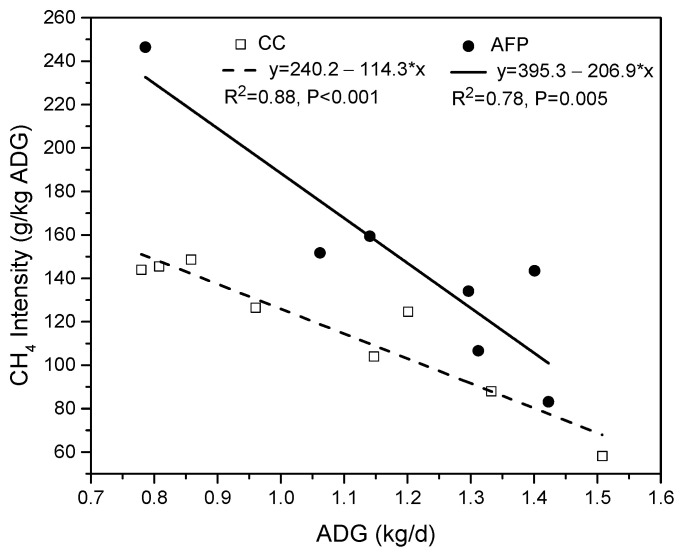
Relationship between methane emission intensity (gCH_4_/kg ADG) and averaged daily gain (ADG) for Hereford steers from CC (white squares) and from AFP (black circles) treatments.

Although no significant differences in emission intensity (gCH_4_/kg ADG) were observed between treatments, Figure 3 indicates a trend of decreasing emission intensity as ADG increases. Typical values of emission intensity range from 110 to 750 gCH_4_/kg ADG [55]. The mean values in this study are close to the lower end of that range, highlighting that the monitored systems are highly efficient in terms of CH_4_ production per unit of product. The advantages of well-managed pastures should be considered, such as higher ADG per hectare and the resulting reduction in emission intensity per unit of ADG produced. Thus, it is essential to strike a balance in utilizing intensive technologies within pasture production systems to enhance forage quality and productivity while minimizing environmental impacts, especially GHG emissions from ruminants [56,57].

The mitigation potential through quality improvement is also reflected in CH_4_ yield, expressed as g CH_4_/kg DMI and Ym (~−30%). In the current experiment, the obtained Ym values fall within the ranges reported in the literature. The CC treatment exhibited a Ym of 4.2%, lower to the IPCC proposed value for well-fed cattle consuming temperate-climate feed types (6.5% ± 1%) [58]. Moreover, the AFP treatment was 6.2%, very close to the mean value suggested. Despite its value, the Ym value does not involve the full range of factors that affect CH_4_ emissions, including digestibility, rumen fermentation characteristics, nutrient profiles, microbial community structure, diet composition, and cattle management practices [59]. Studies conducted in Argentina on pasture-fed beef cattle have variable Ym values, ranging from 4.3% to 8.2% [45]. Moreover, previous research in Uruguay has been determined between 4.2% and 7.9% for beef steers, depending on their diet quality for the winter and spring periods, respectively [60]. The wide range of variation indicates a certain heterogeneity in CH_4_ emissions within the region.

There is a limited number of existing studies that have comprehensively assessed the climate change mitigation benefits of integrate practices that possess CH_4_ mitigation opportunities within the existing traditional system pasture species. Integrated crop–livestock systems have gained interest in recent years due to their benefits in increasing diversification, enhancing soil fertility, and boosting carbon sequestration through the direct return of manure to the soil [61,62]. These systems are widespread globally and provide several economic advantages, such as reduced costs for transporting feed and manure, lower labor demands, and decreased manure storage expenses [63]. Additionally, this study highlights the potential of these systems as a mitigation strategy for enteric CH_4_ emissions. It offers valuable information for estimating carbon balances, including the contribution of livestock, making it an important tool for informed decision-making.

The use of integrated crop and livestock systems to enhance both climate change adaptation and mitigation capacities is becoming increasingly relevant in the southern region of Latin America. De Souza Filho et al. [64] found that southern Brazil has the potential to achieve 22–25% of the target for enteric fermentation emission reduction from the livestock sector, as pledged by the Brazilian government in the Paris Agreement. They concluded that adequate grazing management is a key strategy for improving animal production and reducing the environmental impact of livestock within integrated crop and livestock systems.

## 4. Conclusions

Incorporating high-quality forages into grazing systems has the potential to reduce enteric CH_4_ emissions from grazing steers. This study demonstrates that using cover crop mixtures, including vetch, in cattle diets can lower CH_4_ emissions by improving forage quality. Additionally, these findings provide valuable insights into carbon balance estimation, underscore the role of livestock in emission dynamics, and serve as an essential resource for informed decision-making. Nevertheless, further research is needed to fully understand the mechanisms behind the observed differences in CH_4_ emissions across treatments.

## Figures and Tables

**Table 1:** Forage mass production and botanical composition for each treatment: cover crop (CC) and alfalfa–fescue pasture (AFP).

		Treatments		SEM	*p* Value
		CC	AFP		
Forage production					
Offered forage	DM %	15.7	32.4	0.9	0.006
	Kg DM/ha	1965	1701	134.3	0.298
Residual forage	DM %	15.5	33.0	0.8	0.004
	Kg DM/ha	441.6	396.7	32.7	0.434
Forage use efficiency	%	77.2	76.2	3.7	0.869
Botanical composition offered forage (%)					
Annual ryegrass		26.0	-	3.1	-
Hairy vetch		28.5	-	4.6	-
Forage radish		42.0	-	6.4	-
Alfalfa		-	22.9	9.3	-
Tall fescue		-	49.3	9.2	-
Weeds		1.7	1.1	1.1	0.235
Dead material		1.8	26.7	1.2	0.006

DM: dry matter. SEM: standard error of the mean.

**Table 2:** Chemical composition for the offered forage mass for each treatment: cover crop (CC) and alfalfa–fescue pasture (AFP).

	CC	AFP	SEM	*p* Value
CP (%)	24.3	17.3	1.2	0.050
NDF (%)	36.2	52.6	2.1	0.032
ADF (%)	19.3	31.2	1.8	0.041
DMD (%)	73.8	64.6	1.4	0.041
Ash	12.9	9.4	0.4	0.027
GE (Kcal/Kg DM)	4304.7	4357.7	37.5	0.423

CP: crude protein; NDF: neutral detergent fiber; ADF: acid detergent fiber; DMD: dry matter digestibility; GE: gross energy; SEM: standard error of the mean.

**Table 3:** Dry matter intake, average daily gain, and enteric CH_4_ production of Hereford steers for cover crop (CC) and alfalfa–fescue pasture (AFP) treatments.

	Treatments		EEM	*p* Value
	CC	AFP		
DMI (kg/d)	9.0	9.2	1.2	0.920
ADG (kg/d)	1.1	1.3	0.1	0.334
CH_4_ emissions				
CH_4_ (g/d)	119.1	167.1	9.0	0.002
CH_4_ (g/kg DMI)	13.7	20.4	1.5	0.011
CH_4_ intensity (g/kg ADG)	117.4	146.8	12.6	0.210
Ym (%)	4.3	6.2	0.5	0.013

DMI: dry matter intake. ADG: average daily gain. Ym: methane yield as a percentage of the ingested gross energy.

## Data Availability

The raw data supporting the conclusions of this article will be made available by the authors on request.

## References

[1] Masson-Delmotte V., Zhai P., Pirani A., Connors S.L., Péan C., Berger S., Caud N., Chen Y., Goldfarb L., Gomis M.I. (2021). IPCC Climate Change. The Physical Science Basis. Contribution of Working Group I to the Sixth Assessment Report of the Intergovernmental Panel on Climate Change.

[2] Morgavi D.P., Cantalapiedra-Hijar G., Eugène M., Martin C., Noziere P., Popova M., Ortigues-Marty I., Muñoz-Tamayo R., Ungerfeld E.M. (2023). Reducing enteric methane emissions improves energy metabolism in livestock: Is the tenet right?. Animal.

[3] Islam M., Lee S.S. (2019). Advanced estimation and mitigation strategies: A cumulative approach to enteric methane abatement from ruminants. J. Anim. Sci. Technol..

[4] Johnson K.A., Johnson D.E. (1995). Methane emissions from cattle. J. Anim. Sci..

[5] INDEC (2021). Instituto Nacional de Estadística y Censos. Censo Nacional Agropecuario 2018: Resultados Definitivos.

[6] Bertin O.D., Carrete J.R., Scheneiter J.O., Basail J. (1999). Producción de forraje y de carne, y su resultado económico en pasturas de festuca alta y leguminosas. Pergamino. Estación Experimental Agropecuaria. Rev. Tecnol. Agropecu..

[7] Fernández R., Furch N.E., Bissolino M., Frasier I., Scherger E.D., Quiroga A.R. (2020). Efecto de las pasturas perennes en la fertilidad física y biológica en molisoles de la región semiárida pampeana. Cienc. Suelo.

[8] Wittwer R.A., Dorn B., Jossi W., van der Heijden M.G. (2017). Cover crops support ecological intensification of arable cropping systems. Sci. Rep..

[9] Liu Q., Sun X., Wu W., Liu Z., Fang G., Yang P. (2022). Agroecosystem services: A review of concepts, indicators, assessment methods and future research perspectives. Ecol. Indic..

[10] Abagandura G.O., Sekaran U., Singh S., Singh J., Ibrahim M.A., Subramanian S., Owens V.N., Kumar S. (2020). Intercropping kura clover with prairie cordgrass mitigates soil greenhouse gas fluxes. Sci. Rep..

[11] Alluvione F., Bertora C., Zavattaro L., Grignani C. (2010). Nitrous oxide and carbon dioxide emissions following green manure and compost fertilization in corn. Soil Sci. Soc. Am. J..

[12] Poeplau C., Don A. (2015). Carbon sequestration in agricultural soils via cultivation of cover crops—A meta-analysis. Agric. Ecosyst. Environ..

[13] Restovich S.B., Andriulo A.E., Armas-Herrera C.M., Beribe M.J., Portela S.I. (2019). Combining cover crops and low nitrogen fertilization improves soil supporting functions. Plant Soil.

[14] Rimski-Korsakov H., Alvarez C.R., Lavado R.S. (2015). Cover crops in the agricultural systems of the Argentine Pampas. J. Soil Water Conserv..

[15] Daryanto S., Fu B., Wang L., Jacinthe P.A., Zhao W. (2018). Quantitative synthesis on the ecosystem services of cover crops. Earth Sci. Rev..

[16] Restovich S.B., Andriulo A.E., Portela S.I. (2022). Cover crop mixtures increase ecosystem multifunctionality in summer crop rotations with low N fertilization. Agron. Sustain. Dev..

[17] Beck P.A., Hubbell D.S., Hess T.W., Wilson K.D., y Williamson J.A. (2017). Effect of a forage-type soybean cover crop on wheat forage production and animal performance in a continuous wheat pasture system. Prof. Anim. Sci..

[18] Boval M., Edouard N., Sauvant D. (2015). A meta-analysis of nutrient intake, feed efficiency and performance in cattle grazing on tropical grasslands. Animal.

[19] Beauchemin K.A., Kreuzer M., O’mara F., McAllister T.A. (2008). Nutritional management for enteric methane abatement: A review. Aust. J. Exp. Agric..

[20] Phelan P., Moloney A.P., McGeough E.J., Humphreys J., Bertilsson J., O’Riordan E.G., O’Kiely P. (2015). Forage legumes for grazing and conserving in ruminant production systems. Crit. Rev. Plant Sci..

[21] AOAC (1990). International Association of Official Analytical Chemists.

[22] AACC—American Association of Cereal Chemists (1995). Aproved Methods of the AACC.

[23] Van Soest P.J., Robertson J.B., Lewis B.A. (1991). Methods for Dietary Fiber, Neutral Detergent Fiber, and Nonstarch Polysaccharides in Relation to Animal Nutrition. J. Dairy Sci..

[24] Rohweder D.A., Barnes R.F., Jorgensen N. (1978). Proposed hay grading standards based on laboratory analyses for evaluating quality. J. Anim. Sci..

[25] Johnson K., Huyler M., Westberg H., Lamb B., Zimmerman P. (1994). Measurement of methane emissions from ruminant livestock using a sulfur hexafluoride tracer technique. Environ. Sci. Technol..

[26] Gere J., Gratton R. (2010). Simple, low-cost flow controllers for time averaged atmospheric sampling and other applications. Lat. Am. Appl. Res..

[27] Pinares-Patiño C., Gere J., Williams K., Gratton R., Juliarena P., Molano G., MacLean S., Sandoval E., Taylor G., Koolaard J. (2012). Extending the Collection Duration of Breath Samples for Enteric Methane Emission Estimation Using the SF6 Tracer Technique. Animals.

[28] Gere J.I., Bualo R.A., Perini A.L., Arias R.D., Ortega F.M., Wulff A.E., Berra G. (2021). Methane emission factors for beef cows in Ar-gentina: Effect of diet quality. N. Z. J. Agric. Res..

[29] Short F.J., Gorton P., Wiseman J., Boorman K.N. (1996). Determination of titanium dioxide added as an inert marker in chicken digestibility studies. Anim. Feed Sci. Technol..

[30] Titgemeyer E.C., Armendariz C.K., Bindel D.J., Greenwood R.H., Löest C.A. (2001). Evaluation of titanium dioxide as a digestibility marker for cattle. J. Anim. Sci..

[31] Corbett J.L., Freer M., Jarrige R., Ruckebusch Y., Demarquilly C., Farce M.H., Journet M. (1995). Ingestion et digestion chez les ruminants au pâturage. Nutrition des Ruminants Domestiques, Ingestion et Digestion.

[32] Schalla A., Meyer L., Meyer Z., Onetti S., Schultz A., Goeser J. (2012). Hot topic: Apparent total-tract nutrient digestibilities measured commercially using 120-hour in vitro indigestible neutral detergent fiber as a marker are related to commercial dairy cattle per-formance. J. Dairy Sci..

[33] Balzarini M., Casanoves F., Di Rienzo J.A., González I.A., Robledo C.W., Tablada M.E. (2001). Software Estadístico INFOSTAT.

[34] Inyang U., Vendramini J.M., Sellers B., Silveira M.L.A., Lunpha A., Sollenberger L.E., Adesogan A., Paiva L.M. (2010). Harvest frequency and stubble height affect herbage accumulation, nutritive value, and persistence of ‘Mulato II’ Brachiariagrass. Forage Grazinglands.

[35] Moyo M., Nsahlai I. (2021). Consequences of increases in ambient temperature and effect of climate type on digestibility of forages by ruminants: A meta-analysis in relation to global warming. Animals.

[36] Marín M.F., Naya H., Espasandin A.C., Navajas E., Devincenzi T., Carriquiry M. (2024). Energy efficiency of grazing Hereford heifers classified by paternal residual feed intake. Transl. Anim. Sci..

[37] Kenny D.A., Fitzsimons C., Waters S.M., McGee M. (2018). Invited review: Improving feed efficiency of beef cattle–the current state of the art and future challenges. Animal.

[38] Hill J.O., Coates D.B., Whitbread A.M., Clem R.L., Robertson M.J., Pengelly B.C. (2009). Seasonal changes in pasture quality and diet selection and their relationship with liveweight gain of steers grazing tropical grass and grass legume pastures in northern Australia. Anim. Prod. Sci..

[39] Nichols P.G.H., Revell C.K., Humphries A.W., Howie J.H., Hall E.J., Sandral G.A., Ghamkhar K., Harris C.A. (2012). Temperate pasture legumes in Australia—Their history, current use, and future prospects. Crop Pasture Sci..

[40] Scheeren F.B., Sartor L.R., Danna M., Kuss F., Paris W., Bianchin A., Andriotti N.M., de Menezes L.F.G. (2024). Energy supplementation of beef steers or inclusion of legumes in temperate pastures in crop-livestock integration area. J. Agric. Sci..

[41] Dini Y., Cajarville C., Gere J.I., Fernandez S., Fraga M., Pravia M.I., Navajas E.A., Ciganda V.S. (2019). Association between residual feed intake and enteric methane emissions in Hereford steers. Transl. Anim. Sci..

[42] Carrell R. (2022). Evaluation of Cover Crops as an Alternative Forage Source for Beef Cattle. Ph.D. Thesis.

[43] Planisich A., Utsumi S.A., Larripa M., Galli J.R. (2021). Grazing of cover crops in integrated crop-livestock systems. Animal.

[44] Bárbaro N., Gere J., Gratton R., Rubio R., Williams K. (2008). First measurements of methane emitted by grazing cattle of the argentinean beef system. N. Z. J. Agric. Res..

[45] González F.A., Cosentino V.R.C., Loza C., Cerón-Cucchi M.E., Williams K.E., Bualo R., Constantino A., Gere J.G. (2024). Inclusion of Lotus tenuis in beef cattle systems in the Argentinian flooding pampa as an enteric methane mitigation strategy. N. Z. J. Agric. Res..

[46] Clark H. (2013). Nutritional and host effects on methanogenesis in the grazing ruminant. Animal.

[47] Shibata M., Terada F. (2010). Factors affecting methane production and mitigation in ruminants. Anim. Sci. J..

[48] Beauchemin K.A., Janzen H.H., Little S.M., McAllister T.A., McGinn S.M. (2011). Mitigation of greenhouse gas emissions from beef production in western Canada—Evaluation using farm-based life cycle assessment. Anim. Feed Sci. Technol..

[49] Chung Y.-H., Mc Geough E.J., Acharya S., McAllister T.A., McGinn S.M., Harstad O.M., Beauchemin K.A. (2013). Enteric methane emission, diet digestibility, and nitrogen excretion from beef heifers fed sainfoin or alfalfa1. J. Anim. Sci..

[50] Thompson L.R., Rowntree J.E. (2020). Invited review: Methane sources, quantification, and mitigation in grazing beef systems. Appl. Anim. Sci..

[51] Gaviria-Uribe X., Bolivar D.M., Rosenstock T.S., Molina-Botero I.C., Chirinda N., Barahona R., Arango J. (2020). Nutritional Quality, Voluntary Intake and Enteric Methane Emissions of Diets Based on Novel Cayman Grass and Its Associations with Two *Leucaena* Shrub Legumes. Front. Vet. Sci..

[52] Dillard S.L., Billman E.D., Soder K.J. (2020). Assessment of forage brassica species for dairy and beef-cattle fall grazing systems. Appl. Ani. Sci..

[53] Lileikis T., Nainienė R., Bliznikas S., Uchockis V. (2023). Dietary Ruminant Enteric Methane Mitigation Strategies: Current Findings, Potential Risks and Applicability. Animals.

[54] Ku-Vera J.C., Jiménez-Ocampo R., Valencia-Salazar S.S., Montoya-Flores M.D., Molina-Botero I.C., Arango J., Gómez-Bravo C.A., Aguilar-Pérez C.F., Solorio-Sánchez F.J. (2020). Role of secondary plant metabolites on enteric methane mitigation in ruminants. Front. Vet. Sci..

[55] Zubieta Á.S., Savian J.V., de Souza Filho W., Wallau M.O., Gómez A.M., Bindelle J., Bonnet O.J.F., de Faccio Carvalho P.C. (2021). Does grazing management provide opportunities to mitigate methane emissions by ruminants in pastoral ecosystems?. Sci. Total Environ..

[56] Sakamoto L.S., Berndt A., Pedroso A.d.F., Lemes A.P., Azenha M.V., Alves T.C., Rodrigues P.H., Corte R.R., Leme P.R., Oliveira P.P.A. (2020). Pasture Intensification in Beef Cattle Production can affect Methane Emission Intensity. J. Anim. Sci..

[57] Berndt A., Tomkins N. (2013). Measurement and mitigation of methane emissions from beef cattle in tropical grazing systems: A perspective from Australia and Brazil. Animal.

[58] Dong H., Mangino J., McAllister T., Hatfield J., Johnson D., Lassey K., de Lima M., Romanovskaya A. (2006). Chapter 10: Emissions from livestock and manure management. IPCC Guidelines for National Greenhouse Gas Inventories, Volume 4.

[59] Min B.R., Lee S., Jung H., Miller D.N., Chen R. (2022). Enteric methane emissions and animal performance in dairy and beef cattle production: Strategies, opportunities, and impact of reducing emissions. Animals.

[60] Santander D., Clariget J., Banchero G., Alecrim F., Simon Zinno C., Mariotta J., Gere J., Ciganda V.S. (2023). Beef steers and enteric methane: Reducing emissions by managing forage diet fiber content. Animals.

[61] Tobin C., Singh S., Kumar S., Wang T., Sexton P. (2020). Demonstrating Short-Term Impacts of Grazing and Cover Crops on Soil Health and Economic Benefits in an Integrated Crop-Livestock System in South Dakota. Open J. Soil Sci..

[62] Russelle M.P., Entz M.H., Franzluebbers A.J. (2007). Reconsidering Integrated Crop-Livestock Systems in North America. Agron. J..

[63] Bouwman L., Goldewijk K.K., Van Der Hoek K.W., Beusen A.H., Van Vuuren D.P., Willems J., Rufino M.C., Stehfest E. (2013). Exploring Global Changes in Nitrogen and Phosphorus Cycles in Agriculture Induced by Livestock Production over the 1900–2050 Period. Proc. Natl. Acad. Sci. USA.

[64] de Souza Filho W., de Albuquerque Nunes P.A., Barro R.S., Kunrath T.R., de Almeida G.M., Genro T.C.M., Bayer C., de Faccio Carvalho P.C. (2019). Mitigation of enteric methane emissions through pasture management in integrated crop-livestock systems: Trade-offs between animal performance and environmental impacts. J. Clean. Prod..

